# 786 Decreased Coagulation Activity in Burn Shock Patients Administered Albumin Supplementation

**DOI:** 10.1093/jbcr/irae036.326

**Published:** 2024-04-17

**Authors:** Taha F Hassan, Merry Mathew, Sai Pranathi Bingi, Thomas B Yeater, Colton Shepherd, Alan Pang, John A Griswold

**Affiliations:** Texas Tech University Health Sciences Center School of Medicine, Lubbock, TX; Texas Tech University Health Sciences Center School of Medicine, Lubbock, TX; Texas Tech University Health Sciences Center School of Medicine, Lubbock, TX; Texas Tech University Health Sciences Center School of Medicine, Lubbock, TX; Texas Tech University Health Sciences Center School of Medicine, Lubbock, TX; Texas Tech University Health Sciences Center School of Medicine, Lubbock, TX

## Abstract

**Introduction:**

Burn shock is a systemic inflammatory response and potentially fatal complication of severe burns. Traditional management of burn shock utilizes fluid resuscitation, which involves the administration of crystalloid or colloid solutions. Commonly used colloid solutions include albumin and plasma. Albumin has recently gained traction as a component of this fluid resuscitation; however, it has demonstrated anti-coagulant activity. It is imperative that we study potential coagulation changes as burn patients are already at risk for various other complications.

**Methods:**

This study aims to determine the effect of albumin administration in resuscitation therapy on coagulation in burn patients. To accomplish this goal, we used electronic medical records to compare the indicators of coagulation change in individuals receiving albumin as a part of resuscitation therapy versus those who are not. We posited that the coagulation activity of burn patients decreased in individuals receiving albumin as a component of fluid resuscitation compared to other solution types. A regression analysis was conducted using data from 245 observations for partial thromboplastin time (PTT) and 253 observations for prothrombin time (PT).

**Results:**

The results indicated that total albumin levels showed a significant positive association with both PTT (β = 0.051, p < 0.01) and PT (β = 0.008, p < 0.01), suggesting an anti-coagulant effect. These findings highlight the potential role of albumin in influencing coagulation parameters. Total albumin was found to be correlated positively to the differences in partial thromboplastin time and prothrombin time, showing that albumin exhibits anti-coagulant activity.

**Conclusions:**

The observed anti-coagulant effect of albumin highlights the importance of careful consideration and monitoring when using colloid solutions in burn management

**Applicability of Research to Practice:**

These results build on existing evidence of albumin exhibiting an anticoagulant effect by binding to antithrombin, inhibiting clotting factors and platelet aggregation. Other studies have also found albumin to decrease coagulation in major surgery and oligemic shock patients. This study confirms the anti-coagulant effect of albumin in severe burn patients. The observed anti-coagulant effect of albumin highlights the importance of careful consideration and monitoring when using colloid solutions in burn management. The results of this study, as well as other studies with similar conclusions, will impact evidence-based guidelines for fluid resuscitation in burn patients.

